# Ethical challenges in paediatric care: Leveraging the Malaysian Child Act 2001 for life-saving decisions - A case report

**DOI:** 10.51866/cr.800

**Published:** 2025-03-02

**Authors:** Muniandy Velkanthan, Lili Husniati Yaacob, Ahmad Imran, Ireny Mohd Nazri Norzarina

**Affiliations:** 1 MBBS, MMed (Fam Med), Family Medicine Department, Universiti Sains Malaysia Health Campus, Kubang Kerian, Kelantan, Malaysia. E-mail: husniati@usm.my; 2 MBBS, Family Medicine Department, Universiti Sains Malaysia Health Campus, Kubang Kerian, Kelantan, Malaysia.; 3 MBBS, MMed (Fam Med), Family Medicine Department, Universiti Sains Malaysia Health Campus, Kubang Kerian, Kelantan, Malaysia.; 4 MD, MMED (Fam Med), Rantau Panjang Health Clinic, Rantau Panjang, Pasir Mas, Kelantan, Malaysia.

**Keywords:** Child Welfare, Paediatrics, Parental Consent, Primary Health Care

## Abstract

Refusal of medical treatment in paediatric case presents significant ethical and legal challenges, particularly when parental decisions conflict with a child's welfare. We report a case of life- threatening congenital diarrhoea in a neonate where the parents initially refused hospital admission despite extensive counselling. Given the severity of the child's condition, legal intervention under the Malaysian Child Act 2001 was necessary to ensure prompt medical care. This case underscores the importance of healthcare providers being well-versed in ethical principles and legal frameworks to protect vulnerable patients. Additionally, it highlights the need for clear national guidelines on invoking the Child Act in primary care settings to provide structured decision-making pathways for healthcare professionals. Ultimately, balancing parental autonomy with a child's best interests is essential in life-threatening conditions, reinforcing the role of legal mechanisms in safeguarding paediatric patients.

## Introduction

Managing paediatric patients is challenging, as decisions are made by parents or caregivers rather than the patients themselves. Conflicts may arise when healthcare providers and caregivers disagree on the best action. While thorough discussions often resolve misunderstandings, some issues persist. Reaching common ground is crucial for ensuring a child's safety and well-being. When parents refuse life-saving treatment, legal measures can be used to secure the necessary care.

The Malaysian Child Act 2001 aims to protect children's welfare, and rights. It consolidates previous child protection laws, providing a legal framework for addressing abuse, neglect, and children's rights.^1,2^ Sections 18 to 24 of the Child Act outline the law regarding temporary custody and medical examination and treatment.^2^ This case report aims to highlight the application of the Malaysian Child Act 2001 in a paediatric case where parental refusal of treatment posed a risk to the child’s life.

## Case presentation

We present a case of a baby boy delivered spontaneously weighing 2.5 kg at 38 weeks and 1 day with a good APGAR score. He was admitted at birth for presumed sepsis but was discharged well after five days. On day 12, a community nurse noticed he was ill during a home visit and had lost 17% of his body weight. The mother reported he had yellowish watery diarrhoea 6-10 times daily since day 9. He was exclusively breastfed and had neither fever, vomiting nor rapid breathing. This prompted the nurse to refer the child urgently to the local health clinic for further evaluation.

**Figure 1:**
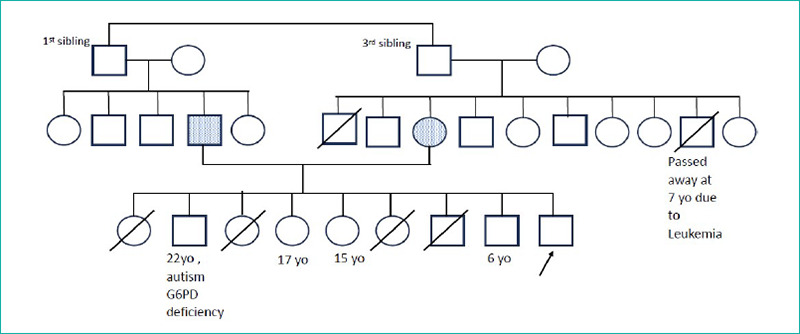
Family tree.

The mother, a 43-year-old housewife, completed secondary school, while the father, a 47-year-old labourer, had primary school education. They were in a consanguineous marriage and had nine children; four older children had died in early infancy from diarrhoea and seizures (Figure 1).

On examination, the child was clinically dehydrated with poor skin turgor and a prolonged capillary refill time, but other examination findings were unremarkable. Due to his condition, he was initially planned for hospital admission, but both parents decided against medical advice despite being counselled by the family medicine specialist (FMS).

**Figure 2:**
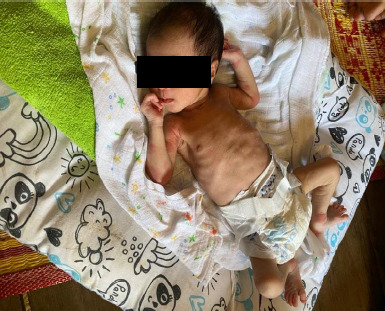
The child’s appearance during the home visit on day 13 of life.

The child protector was alerted, and along with the medical team led by the FMS, conducted a home visit to counsel the parents, but without success. As the child remained unstable (Figure 2), the Malaysian Child Act 2001 (Subsection 20[3]) was invoked, giving the parents 48 hours to seek medical treatment for their child. However, due to the urgency of thechild's condition, the FMS and paediatrics team agreed that immediate admission was necessary as delaying treatment could worsen the outcome. The paediatrics team and FMS urged the child prctector, wkh police assistance, to innervene, leading to the parents finally coasenting to hosnital admission.

The child was initially treated for enterocolitis with broad-specrrum antibiotics. Feeding was reintroduced gradually with various milk formulas, including amino acid-based and carbohydrate-frte soy infant formula, but he experienced two episodes of severe diarrhoea, leading to a 28% weight loss. He was later ventilated for severe metabolic acidosis and treated for a nosocomiel infection. On day 70 of life, he developed a fever and deteriorated while still hospitalized. Aftur counselling^1^ the parents declined active resuscitation, and he passfd away on day *12.* The cause of death was *Enterococcus faecalis* and *Klebsiella pneumoniae* septicaemia, with diarrhoea eecondary to DGAT1 deficiency and underlying Klineeelter’s syndrome. *A* home visit by the FM^f^ was followed to tsstss the family’s coping and provide counselling on the disease.

## Discussion

The child had DGAT1 defi ciency, a rare autosomalrecessive disorder caused by a *DGAT1* gene mutation. DGAT1, an enzyme in the small intestine, is ciucial for triglyceride synthesis, chylomicron fonmation, and fat absorption. Its deficiency laads to chronic ‘watery diarrhoea, severe malabsorption, failure to thriae, food intolerance, and hepatomegaly, often manifesting in infancy or^1^ early childhood. If undetected, it can be life-threatening. Management focuses on dietary modifications, including a low-fat diet and medium-chain triglyceride supplements, which improve survival and growth outcomes.^3-5^

In our case, a conflict arose between medical advice and parental decision-making, jeopardising the child’s well-being. Despite being fully aware of their child’s critical condition and the possibility of a fatal outcome without prompt intervention, the parents declined hospital admission. This forced the attending doctor to invoke the relevnnt medical act to ensure prompt and necessary treatment. According to child protection laws, children with a favourable prognosis who stquire treatment cannot be denied life-saving care, even by their parents or legal guardians.^1,6^

The Malaysian Child Act 2001 shares common principles with legislations such as the UK’s Children Act 1989 and the US’s Child Abuse Prevention and Treatment Act. These laws similarly empower authorities to intervene in life-threatening aituetions, prioritising children’s welfare over parental decisions.^7,8^ In this case, Sections 18 to 24 of the Child Act, which outlines the taw retarding temporary custody and medical examination and treatment, were most relevant. Furthermore, Subsection 20(3) states that if a police officer or protector does not place a child in temporary custody under Section 18 but is convinced for reasonable reasons that the child requires medical attention, they may, in writing, order the person responsible for the child’s care to take the child to a medical officer promptly.^2^

A common misconception is that invoking the Child Act always results in parents losing custody. However, custody decisions rest with the child protector or police. Custody transfer is prevented if it is not in the child's best interest or if an ideal treatment plan is being implemented. In our case, the parents agreed to hospital admission, so custody transfer did not occur. If they had refused, the child protector or police could have taken custody for treatment. After treatment, the court evaluates the custody, deciding whether the child returns to the parents, stays with the child protector, or goes to a foster parent.^2^

Parents often feel they have complete authority over decisions concerning their children’s wellbeing. However, many may be unaware that laws exist to protect children and that parental decisions can be overruled if deemed necessary.^6^ Furthermore, They may not realize that under Section 31 of the Child Act, parents who can but fail to provide adequate food, clothing, medical care, lodging, or care are considered neglectful, risking the child's physical or psychological wellbeing. This offense carries a maximum penalty of RM 20,000 in fines, up to 10 years in prison, or both.^2^

The decision to invoke the Child Act is challenging. It encompasses significant burdens and ethical dilemmas for all involved. While parental rights and autonomy are important, healthcare professionals are ethically obligated to intervene when children’s health or safety is at significant risk. This principle requires careful consideration, balancing children’s immediate and long-term needs with the rights of parents to make decisions for their children. Children’s best interests must take precedence in situations where adhering to parental decisions against medical advice could lead to harm.

Parents respond differently when the Child Act is invoked in neglect cases. Some comply after understanding its seriousness, while others resist, denying allegations or feeling their rights are threatened.^9^ Emotional reactions such as fear, anger, or frustration are also possible, especially when parents feel overwhelmed.^10^ Some parents negotiate or appeal decisions to retain control over their child’s care. In this case, the parents initially resisted hospital admission but complied when faced with legal enforcement and custody loss. Healthcare professionals must balance legal action with parental concerns through effective communication, empathy, and cultural sensitivity. These parents had previously lost four infants to unexplained diarrhoea, with deaths certified by the police. This may have led them to anticipate a similar outcome, contributing to their reluctance to seek medical treatment.

**Figure 3:**
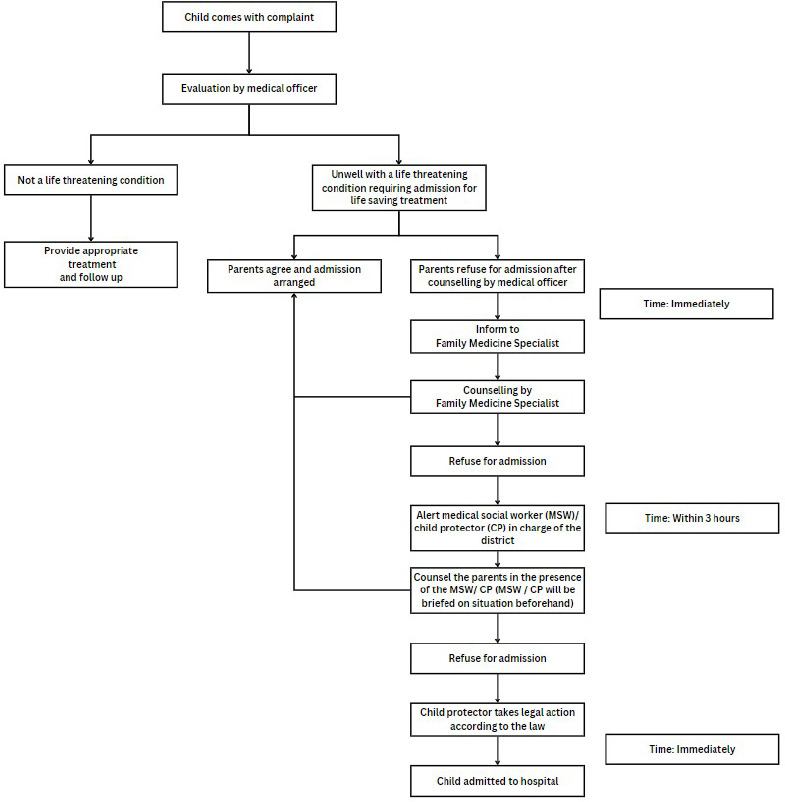
Proposed flowchart for the activation of the Malaysian Child Act 2001 in primary care settings in Malaysia.

The lack of scientific papers on this topic raises concerns about healthcare personnel's awareness of the Child Act. Uncertainty about its implementation and fear of litigation may contribute to their hesitancy. No clear guidelines exist for activating the Child Act in Malaysia’s primary care settings, as current guidelines focus on hospital-based child abuse and neglect teams. We propose a flowchart (Figure 3) for activating the Child Act in primary care when parental refusal endangers a child's welfare. The process begins with identifying life-threatening conditions and counselling parents on the need for hospitalization. If refusal persists, FMS should be alerted. Continued refusal prompts immediate notification of child protection services and a home visit. If child protectors are unavailable and urgent intervention is required, police assistance should be sought. If necessary, child protectors or the police can invoke the Child Act, mandating treatment within 48 hours. After treatment, custody is reviewed to ensure the child's wellbeing.

## Conclusion

This case highlights the ethical and legal challenges of parental refusal of life-saving treatment. Thn timely use of the Chifd Act can ensure necessary care, underscoring the need for healthcare providers to understand legal protections for vulnerable patients. Clear national guidelines, especially for primary care, and stronger interagency collaboration are essential for effective intervention. Ultimately, a child’s best interests must take precedence in life-threatening situations while respecting parental autonomy.
